# Ultralow Laser Power Three-Dimensional Superresolution Microscopy Based on Digitally Enhanced STED

**DOI:** 10.3390/bios12070539

**Published:** 2022-07-20

**Authors:** Xiaochun Shen, Luwei Wang, Wei Li, He Wang, Hanqiu Zhou, Yinru Zhu, Wei Yan, Junle Qu

**Affiliations:** Shenzhen Key Laboratory of Photonics and Biophotonics, Key Laboratory of Optoelectronic Devices and Systems of Ministry of Education and Guangdong Province, College of Physics and Optoelectronic Engineering, Shenzhen University, Shenzhen 518060, China; 2100453080@email.szu.edu.cn (X.S.); wanglowell@szu.edu.cn (L.W.); liwei_98forward@163.com (W.L.); wangh3016@163.com (H.W.); zhouhanqiu6666@163.com (H.Z.); 2150453014@email.szu.edu.cn (Y.Z.); jlqu@szu.edu.cn (J.Q.)

**Keywords:** stimulated emission depletion, 3D super-resolution imaging, digital enhancement, low power STED

## Abstract

The resolution of optical microscopes is limited by the optical diffraction limit; in particular, the axial resolution is much lower than the lateral resolution, which hinders the clear distinction of the three-dimensional (3D) structure of cells. Although stimulated emission depletion (STED) superresolution microscopy can break through the optical diffraction limit to achieve 3D superresolution imaging, traditional 3D STED requires high depletion laser power to acquire high-resolution images, which can cause irreversible light damage to biological samples and probes. Therefore, we developed an ultralow laser power 3D STED superresolution imaging method. On the basis of this method, we obtained lateral and axial resolutions of 71 nm and 144 nm, respectively, in fixed cells with 0.65 mW depletion laser power. This method will have broad application prospects in 3D superresolution imaging of living cells.

## 1. Introduction

Cells are the smallest functional and structural unit of living organisms and understanding their 3D structure at the nanoscale is of great significance to biomedicine. Currently, the optical microscope is the most efficient tool for studying the 3D structure of cells, but due to the existence of the optical diffraction limit, the lateral and axial resolutions of traditional optical microscopes are approximately 200 nm and 500 nm, respectively, and both of these sizes are much larger than many subcellular structures [1]. Therefore, 3D superresolution optical imaging technology is necessary to study the 3D structure of cells. In recent decades, scientists have proposed several superresolution microscopy (SRM) imaging techniques based on fluorescence switching effects and single-molecule localization to break the optical diffraction limit [2,3,4,5] and improve the resolution, which have helped to reveal the details of subcellular organelles and provided a technical basis for further research in cell biology [6,7,8,9]. STED microscopy is a purely optical method based on confocal microscopy to break the diffraction limit and achieve superresolution imaging [2,10,11,12], while it has the advantages of high spatial resolution, large imaging depth, and 3D superresolution imaging. STED utilizes two laser beams: An excitation beam to generate fluorescence through spontaneous emission and a donut-shaped depletion beam (STED beam) to suppress the fluorescence at the periphery of the excitation beam so that only the center of the excitation beam can emit fluorescence to achieve superresolution imaging [13,14,15].

In living cells, using a high laser power is not advisable as it can cause severe photodamage in biological samples and photobleaching of fluorophores to hinder long-term STED imaging. Therefore, reducing the need for depletion power is significant for further developments in biological research. In recent years, scientists have made many efforts to improve imaging quality and spatial resolution at a low depletion power. For instance, in 2013, S. W. Hell’s group proposed a time-gated detection approach [16,17,18] that excludes photons that deplete light regions by selecting long-lifetime photons. In 2013, Kuang et al. proposed the fluorescence emission difference (FED) [19], a superresolution imaging method similar to STED. FED uses a confocal image minus a negative confocal image to obtain a high spatial resolution at low depletion power. In 2017, Gao et al. proposed the background suppression approach, namely, STEDD [20], which adopts double depletion to remove a fraction of fluorophores in peripheral regions of incomplete depletion or re-excitation by the STED beam. Our research group also developed a series of ways to reduce the demand for depletion power. For example, in 2017, coherent optical adaptive techniques were introduced into STED for the first time, which effectively improved the imaging depth and spatial resolution [21]. In 2018, a STED-FLIM system was established based on phasor plot analysis [22], which successfully separated photons of different lifetimes without increasing the depletion power. In particular, in 2020, our research group proposed digitally enhanced STED (DE-STED) technology, which can achieve a resolution of ~λ/8 under ultralow depletion power [23,24,25,26].

Although these technologies have effectively relaxed the high depletion power demand of STED superresolution imaging, few reports are available on reducing laser power in the field of 3D superresolution imaging. Currently, the main methods for achieving 3D-STED superresolution imaging are noncoherent wavefront modulation [27] and a 4π 3D-STED system [28,29,30]. Noncoherent wavefront modulation is based on a single objective lens architecture; through ring phase plate angles of 0~π and spiral phase plate angles of 0~2π on the two depleted light space modulations, a lateral and an axial donut-shaped beam can be produced so that the two incoherent lights coincide to obtain a 3D hollow STED beam. The 4π 3D-STED system is based on a two-objective lens architecture in which the depleted light is interfered with by two opposing objectives on the sample surface to form a hollow spherical spot. Compared with the single-objective architecture, this method has obvious advantages in resolution, but the alignment requirements of the optical path of the system are strict, and the system complexity is high. In 2016, Yang et al. [31] placed a mirror behind a sample to make the incident light and reflected light interfere with the focal plane of the sample. In 2018, Xue et al. [32] built a 3D-STED system based on structured light. Although these 3D STED methods can reduce costs and system complexity, they still face the problem of excessive demand for depletion laser power.

Here, we introduce a new ultralow laser power 3D superresolution imaging method based on DE-STED, namely, 3D DE-STED, which can be applied to any 3D STED microscope without modifying the optical system. In this study, we combined the digital enhancement method [23] with the commercial Leica SP8 system to achieve low-power 3D superresolution imaging. In the 3D DE-STED method, digital enhancement can be performed simultaneously laterally and axially. First, a 3D hollow ellipse image is obtained by subtracting the STED image from the confocal image. Then, the hollow ellipse image is digitally enhanced. The 3D DE-STED image is obtained by subtracting the digitally enhanced 3D hollow ellipse image from the original confocal image multiplied by an enhancement coefficient. This approach can greatly reduce the power required for STED beams, making it possible to perform long-term superresolution imaging of living cells in 3D. The effectiveness of this method is demonstrated in fluorescent beads and nuclear pore complexes, featuring superior-quality final images.

## 2. Materials and Methods

All confocal and STED images were obtained by a commercial STED microscope (Leica SP8 STED 3X, TCS SP8 STED 3X, Leica Microsystems, Wetzlar, Germany). For detailed parameters of the image acquisition process, see Section S1 of the Supporting Information. All laser powers were measured at the front aperture of the objective (HC PL APO CS2 100x/1.40 OIL, Leica, Wetzlar, Germany). The fluorescence signal was recorded with a sensitive detector (Leica HyD, Leica, Wetzlar, Germany. The images were deconvoluted with Huygens essential software (Scientific Volume Imaging) to increase the image contrast, while detailed parameters of the deconvolution process are provided in Section S2 of the Supporting Information.

**Figure 1:**
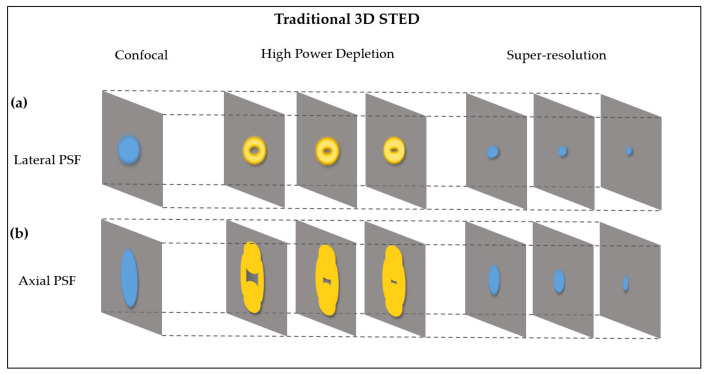
PSF of 3D STED microscopy. (**a**) Laterally, the donut-shaped STED beam suppresses lateral peripheral fluorescence. (**b**) Axially, the bottle-like STED beam suppresses axial peripheral fluorescence.

**Figure 2:**
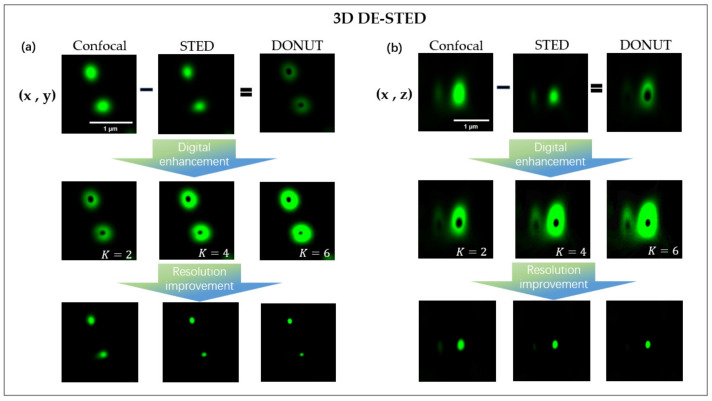
Schematic principles of 3D DE-STED. (**a**) Schematic of the distribution of PSF in (x, y). (**b**) Schematic of the distribution of PSF in (x, z). The center zero-intensity area of the 3D hollow exhaustion spot decreases with increasing K. The principle of 3D DE-STED for achieving resolution improvement in the sample of fluorescent beads with increasing K and without increasing the depletion laser power.

In traditional 3D STED imaging (Figure 1), on the lateral side, the lateral (point spread function) PSF is suppressed by a donut-shaped STED beam. On the axial side, the axial PSF is suppressed by a bottle-like STED beam. The zero-intensity regions of the lateral and axial STED beams decrease as the depletion power increases. B By superimposing donut-shaped and bottle-like beam PSFs on the excitation PSF, the resolution can be enhanced on 3D spatial planes to achieve 3D-STED superresolution images. In the 3D DE-STED, the STED image is subtracted from the confocal image to generate a 3D depleted hollow spot image, and the confocal and STED images are obtained from the same imaging position. The digital enhancement method can be used to realize the process of increasing the depletion power in the traditional STED and improving the depletion efficiency. We multiply the resulting 3D hollow exhaustion spot by the K coefficient (greater than 1), and the zero-intensity area in the center of the hollow depleted spot decreases with the increase in K (Figure 2), which is equivalent to enhancing the power of the depletion laser. After subtracting the enhanced depleted spot image from the original confocal image, a 3D DE-STED image with a higher resolution can be obtained, achieving a resolution increase in three-dimensional space.

The detailed mathematical formula of 3D DE-STED imaging is presented in Section S3 of the Supporting Information. When the intensity of pixels in the enhanced donut image is greater than that in the confocal image, negative values appear in the DE-STED image during the process of image subtraction. This indicates that the saturated stimulated emission has been reached, just as in the case of high-power STED imaging. Because the DE-STED image is derived from the confocal image, DE-STED should be between the maximum and minimum values of confocal. Therefore, the negative values can be clipped to zero without the loss of superresolution signals from the very center of the excitation laser spot [19,23,33].

The core of the 3D DE-STED method is to increase the intensity of the donut PSF by K times to decrease the effective fluorescence area. As long as the center intensity of the donut-shaped depletion beam is maintained at zero, the PSF of the excitation laser spot can be compressed infinitely without duction in peak intensity after subtracting the enhanced donut image. We used the SNR and peak SNR (PSNR) as the specifications to assess the image quality. However, in practice, the peak intensity of the DE-STED PSF is severely reduced due to nonzero intensity in the center of the donut PSF, which leads to the PSNR of the 3D DE-STED image decreasing with K and then stabilizing (Section S4 and Figure S1).

## 3. Results and Discussion

### 3.1. 3D DE-STED Imaging of Fluorescence Beads

#### 3.1.1. Imaging of Fluorescent Beads on the Lateral Profile

The spatial resolution of 3D DE-STED microscopy was evaluated by measuring the lateral and axial PSFs of 100 nm fluorescent beads provided by Abberior. The fluorescent beads were labeled with Abberior STAR 488 dye. 

**Figure 3:**
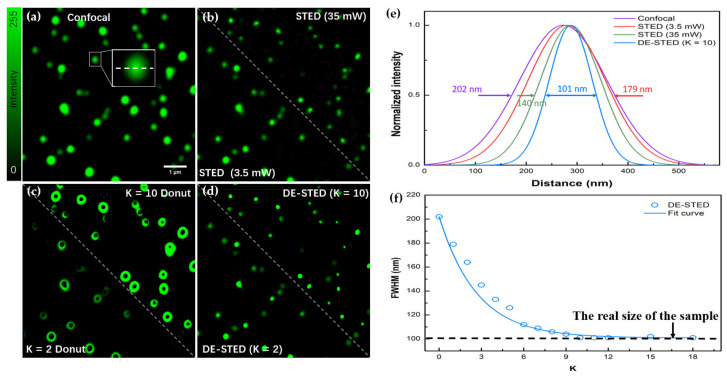
DE-STED imaging results for fluorescent beads on the lateral profile. (**a**) Confocal image. (**b**) STED image with depletion powers of 3.5 and 35 mW. (**c**) Donut image, where K = 2 and 10. (**d**) DE-STED results at K = 2 and 10. (**e**) FWHM curves for confocal, STED (3.5 and 35 mW), and K = 10 DE-STED, taking the marked fluorescent beads in (**a**) as the research object. (**f**) Curves of the relationship between the FWHM and the K value. Field of view (FOV): 7.43 μm. Scale bar: 1 μm.

Figure 3a,b,d shows confocal, STED, and DE-STED images of fluorescent beads on the lateral side, respectively. The excitation light wavelength was 505 nm, and the depletion light wavelength was 592 nm. Figure 3e shows that when the depletion laser power was 3.5 mW, the full width at half maximum (FWHM) of confocal microscopy and STED was 202 and 179 nm, respectively. Because of the low depletion power, the increase in resolution was not noticeable. When the depletion power increased to 35 mW, the FWHM dropped to 140 nm.

As is well known, higher depletion powers result in smaller FWHMs. Therefore, to obtain a perfect resolution, in traditional STED, the demand for depletion is very powerful. However, higher depletion power not only results in a reduced signal-to-noise ratio but also increases photobleaching. To obtain the DE-STED image, we subtract the STED image (Figure 3b) from the confocal image (Figure 3a) and then obtain the donut image (Figure 3c). The DE-STED image (Figure 3d) is composed of the confocal image (Figure 3a) minus the donut image (Figure 3c), where K is 2 and 10. Because a DE-STED image with a K value of 1 is a raw STED image, an increase in resolution can only be achieved if the enhancement coefficient is greater than 1.

As Figure 3f shows, the resolution of fluorescent beads increases significantly with an increasing K value. When K = 10 at 3.5 mW depletion laser power, the FWHMs of the DE-STED image are 101 nm, which is higher than the STED image resolution at 35 mW of STED power and close to the real size of the fluorescent bead itself. Because of the actual size limitations of the beads, when K = 10, the resolution stops increasing as the K value increases.

#### 3.1.2. Imaging of Fluorescent Beads on 3D

**Figure 4:**
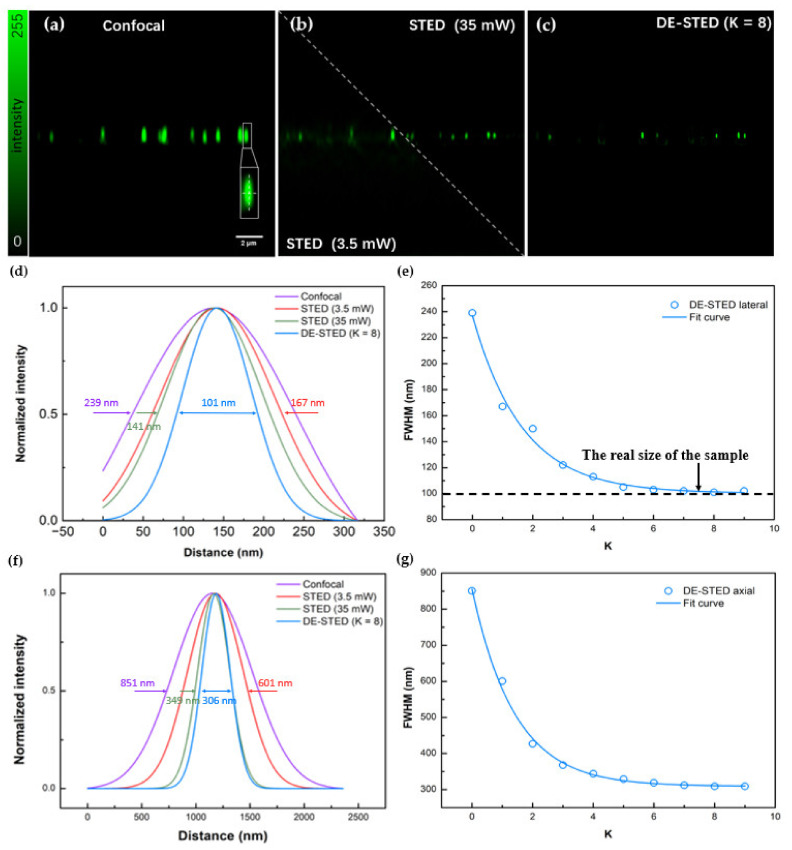
3D DE-STED imaging results for fluorescent beads on the axial profile. (**a**) Confocal image. (**b**) STED image with depletion powers of 3.5 and 35 mW. (**c**) DE-STED results at K = 8. (**d**,**f**) Lateral and axial FWHM curves for confocal, STED (3.5 and 35 mW), and K = 8 DE-STED, taking the marked fluorescent beads in (**a**) as the research object. (**e**,**g**) Curves of the lateral and axial relationship between the FWHM and the K value. Field of view (FOV): 18.03 μm. Scale bar: 2 μm.

The same results were observed on the axial plane. Figure 4a–c shows confocal, STED, and DE-STED images of fluorescent beads on the axis, respectively. When the depletion laser power was 3.5 mW, the lateral FWHMs of confocal and STED were 239 and 167 nm, respectively (Figure 4d), and the axial FWHMs of confocal and STED were 851 and 601 nm, respectively (Figure 4f). When the depletion laser power was 35 mW, the lateral FWHM of STED dropped to 141 nm (Figure 4d), while the axial FWHM of STED dropped to 349 nm (Figure 4f). Treated with the DE-STED method, at K = 8, the lateral FWHM of 3D DE-STED was 101 nm, which was close to the real size of the fluorescent beads (Figure 4e), and the axial FWHM of 3D DE-STED was 306 nm (Figure 4g), which was higher than the resolution at 35 mW STED beam power. As shown in Figure 4e,g, when K was greater than 8, the lateral and axial resolution stopped increasing. The above results show that at a depletion laser power of 3.5 mW, the DE-STED method of K = 8 can obtain a higher resolution than the 35 mW depletion laser power. These results show that three-dimensional DE-STED is a powerful imaging method at ultralow power for achieving higher resolution.

### 3.2. 3D DE-STED Imaging of Fixed Biological Cells

#### 3.2.1. Imaging of the Lateral Nuclear Pore Complex

**Figure 5:**
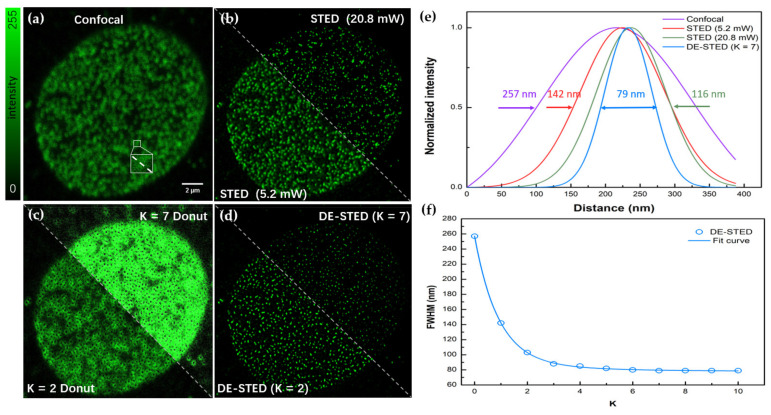
DE-STED imaging results for the nuclear pore complex on the lateral profile. (**a**) Confocal image. (**b**) STED image with depletion powers of 5.2 and 20.8 mW. (**c**) Donut image, where K = 2 and 7. (**d**) DE-STED results at K = 2 and 7. (**e**) The FWHM curves for confocal, STED (5.2 and 20.8 mW), and K = 7 DE-STED, taking the marked white underlined part in (**a**) as the research object. (**f**) Curves of the relationship between the FWHM and the K value. The field of view (FOV) is 20.88 μm, Scale bar: 2 μm.

The ability of 3D DE-STED to improve the resolution at a low depletion power is also valid when imaging subcellular structures. To demonstrate the power of 3D DE-STED, we performed STED imaging of a commercial fixed cells sample. The samples we used for the experiments were HeLa cells provided by the Abberior Company with a thickness of approximately ten microns. In this sample, the nuclear pore complex (NPC) was stained by Abberior STAR RED, and the imaging depth in our experiment was approximately five microns. The DE-STED results on the lateral NPC are shown in Figure 5.

Figure 5a,b,d shows the confocal, STED, and DE-STED images, respectively. Figure 5c shows the donut image obtained by subtracting the STED image of 5.2 mW from the confocal image, including K = 2 and 7. Figure 5e shows that when the 640 nm excitation light was 10 μW and the 775 nm depleted light was 5.2 mW, the FWHMs were 257 and 142 nm for confocal and STED, respectively. The FWHM decreased to 116 nm when the depletion power was increased to 20.8 mW. As Figure 5f shows, when K = 7 at 5.2 mW depletion laser power, the FWHM of the DE-STED image was 79 nm, which is higher than the STED image resolution at 20.8 mW of STED power, and its resolution no longer changes when the K value increases.

#### 3.2.2. Imaging of Nuclear Pore Complexes in 3D

**Figure 6:**
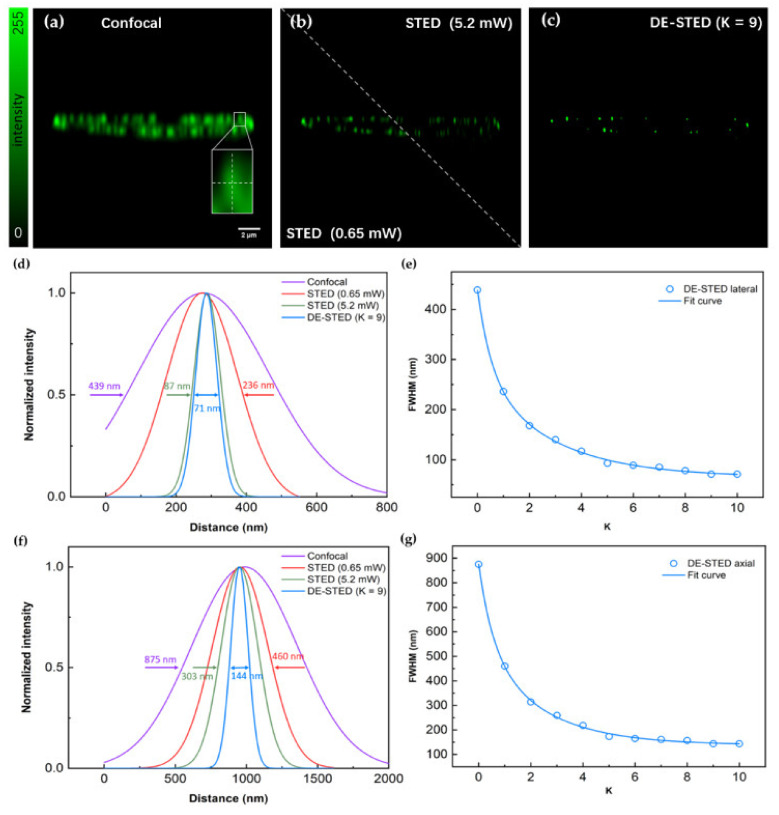
3D DE-STED imaging results for the nuclear pore complex on the axial profile. (**a**) Confocal image. (**b**) STED image with depletion powers of 0.65 and 5.2 mW. (**c**) DE-STED results at K = 9. (**d**,**f**) Lateral and axial FWHM curves for confocal, STED at 0.65 and 5.2 mW, and K = 9 DE-STED, taking the marked white underlined part in (**a**) as the research object. (**e**,**g**) Curves of the lateral and axial relationship between the FWHM and the K value. The field of view (FOV) is 21.78 μm, Scale bar: 2 μm.

The axial images of the nuclear pore complex were then studied. The excitation light wavelength was 635 nm, and the depletion wavelength was 775 nm. Figure 6a,b shows the confocal and STED images of the nuclear pore complex on the axis, respectively. The white frame area is taken as the research object. First, analyzed on the horizontal axis, the FWHM of the confocal image was 439 nm, and the FWHMs of STED were 236 and 87 nm when the depletion power was 0.65 and 5.2 mW, respectively. Although a better resolution was obtained at a high depletion power, the intensity of imaging dropped dramatically and induced irreversible bleaching damage to biological samples.

To address this problem, we performed a DE-STED experiment, and the results are shown in Figure 6c. Figure 6e shows the change in the FWHM on the lateral of DE-STED images at different K values, and the FWHM gradually improves as the K value increases. However, when the K value was greater than 9, the resolution was not further improved and was eventually limited to approximately 71 nm. As shown in Figure 6d, at 0.65 mW STED optical power, the resolution obtained by the DE-STED method of K = 9 on the lateral side exceeds the effect of the STED optical power of 5.2 mW in Figure 6b.

Then, analyzed on the vertical axis, as shown in Figure 6f, the FWHM of the longitudinal axis in the white frame area of the confocal image is 875 nm, the resolution of 0.65 mW STED power is 460 nm, and the resolution of 5.2 mW STED is 303 nm. At low STED optical power, STED images do not have a noticeable superresolution effect. Using the 3D DE-STED method at 0.65 mW STED optical power, Figure 6g shows that as the K value increases, the FWHM gradually decreases, and finally, at K = 9, an FWHM of 144 nm is reached on the vertical axis, and it is no longer reduced, which is better than the resolution of the depletion power at 5.2 mW.

Therefore, with the above results, we observe that at a low STED power of 0.65 mW, lateral and vertical resolution can respond to the DE-STED method. Its optimal K value is 9, and the 3D spatial resolution is finally limited to 71 nm on the lateral axis and 144 nm on the vertical axis. The result can completely replace the results of a high STED power of 5.2 mW.

### 3.3. 3D DE-STED Imaging of Living Cells

**Figure 7:**
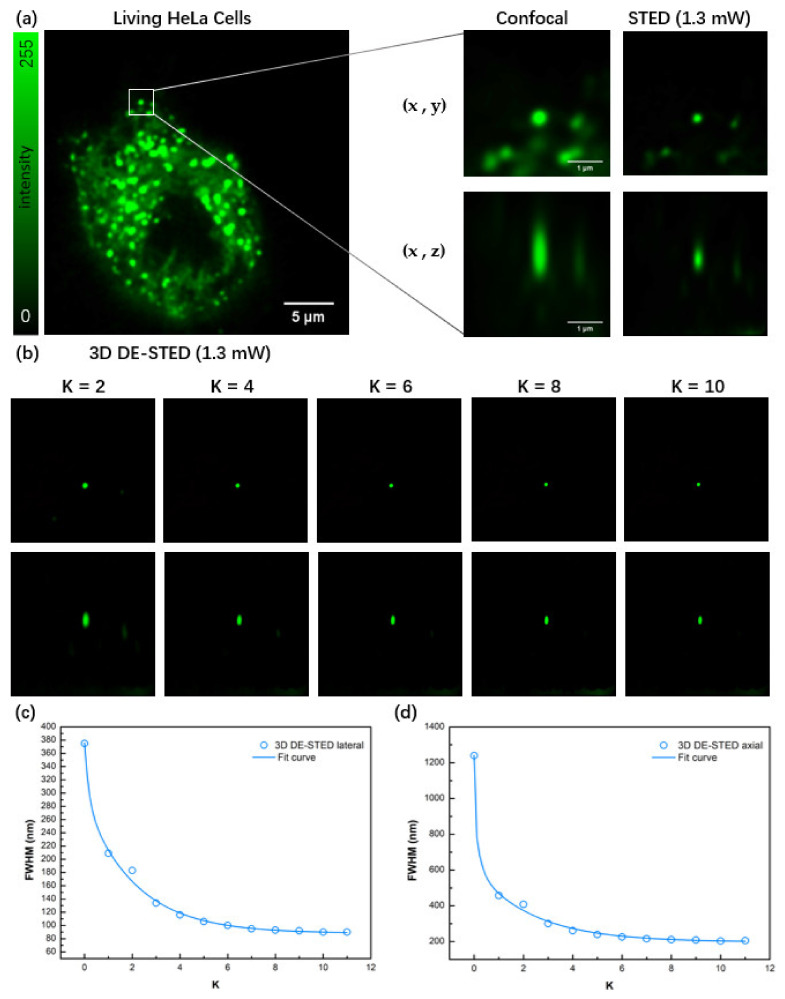
3D DE-STED imaging of lysosomes in living HeLa cells. (**a**) Confocal image of a whole HeLa cell and enlarged confocal and STED images in the white square. (**b**) 3D DE-STED images with the increase in K value. The field of view (FOV) is 4.15 μm. (**c**,**d**) Curves of the lateral and axial relationship between the FWHM and the K value. Its optimal K value is 10.

The advantage of 3D DE-STED is that it can achieve 3D superresolution imaging with low depletion power, which is particularly important in live cell imaging applications. To demonstrate this advantage, Lysosomes of live HeLa cells were labeled with LysoTracker Deep Red (Thermo Fisher, Waltham, MA, USA) and used as the sample. Imaging was performed using the commercial Leica SP8 system with a 100x objective lens, and the excitation light wavelength was 635 nm while the depletion wavelength was 775 nm. The experimental results are shown in Figure 7.

Figure 7a shows the confocal image of a whole HeLa cell and enlarged confocal and STED images in the white square, of which the depletion laser power was 1.3 mW. We used K coefficient values from 2 to 10 to observe the image changes in terms of resolution and intensity, and the results are shown in Figure 7b. Figure 7c,d shows that with a lower depletion power of 1.3 mW, the resolution of the 3D DE-STED image increases substantially with the increase in the K value. However, it does not further improve when the K value is greater than 10 and is eventually limited to approximately 90 nm in the lateral axis and 200 nm in the vertical axis.

Compared with traditional 3D STED, low-power 3D DE-STED shows considerable advantages in terms of improving spatial resolution through the K-coefficient at low depletion power. At low depletion power, the resolution of DE-STED images can be greatly improved with increasing K. Theoretically, using higher depletion power can increase the resolution of DE-STED images with smaller K values, but as the depletion power increases, the signal strength rapidly decreases. Therefore, selecting a low-power STED image for processing when using the 3D DE-STED method is a better choice for obtaining a high signal-to-noise ratio and superresolution images.

## 4. Conclusions

In this paper, we present the ultralow-power 3D DE-STED method. The DE-STED method, which can achieve superresolution imaging at ultralow power, was the previous work of our research group. It achieves a spatial resolution of λ/8 at a STED power of 1.4 mW, which is an order of magnitude lower than that of traditional STED methods. In 3D STED microscopy, photobleaching and resolution are two contradictory obstacles. Therefore, we combine the advantages of the commercial Leica SP8 three-dimensional superresolution imaging system and digital enhancement algorithm, and 3D DE-STED imaging is performed with fluorescent beads of 100 nm, fixed cells, and living cells. In the experiment, by multiplying by different K-coefficients, different resolutions can be flexibly and conveniently obtained. Then, the experimental results show that when K = 9, the 3D DE-STED microscope can obtain a spatial resolution of 71 nm in the lateral axis and 144 nm in the vertical axis at 0.65 mW of depletion power. The advantage of the 3D DE-STED method is that the system does not need to be modified and is flexible. It can reduce the phototoxicity of biological samples and the photobleaching of probes, which has great potential in long-term superresolution live-cell imaging.

## Data Availability

The data presented in this study are available on request from the corresponding author.

## Supplementary Materials

The following supporting information can be downloaded at: https://www.mdpi.com/article/10.3390/bios12070539/s1, Section S1: Image acquisition, Section S2: Deconvolution, Section S3: Mathematical formula of 3D DE-STED imaging, Section S4: Calculation of the SNR and PSNR, Figure S1: The curves of the relationship between PSNR and the K value of fluorescent beads’ 3D DE-STED image.
