# Supplementary material for: Ultralow Laser Power Three-Dimensional Superresolution Microscopy Based on Digitally Enhanced STED
Source: Biosensors (Basel). 2022 Jul 20;12(7):539. doi: 10.3390/bios12070539 (PMC9351679; doi:10.3390/bios12070539)
Supplement: Supplementary file 1 [file biosensors-12-00539-s001.zip › biosensors-1790470-supplementary.pdf]

biosensors

MDPI

Supporting information

Ultralow Laser Power Three-Dimensional Superresolution Microscopy Based on Digitally Enhanced STED

Xiaochun Shen, Luwei Wang, Wei Li, He Wang, Hanqiu Zhou, Yinru Zhu, Wei Yan,* and Junle Qu

College of Physics and Optoelectronic Engineering, Shenzhen Key Laboratory of Photonics and Biophotonics, Key Laboratory of Optoelectronic Devices and Systems of Ministry of Education and Guangdong Province, Shenzhen University, Shenzhen 518060, P. R. China
2100453080@email.szu.edu.cn(X.S.); wanglowell@szu.edu.cn(L.W.); liwei_98forward@163.com(W.L.); wangh3016@163.com (H.W); zhouhanqiu6666@163.com (H.Z); 2150453014@email.szu.edu.cn(Y.Z); jlqu@szu.edu.cn (J.Q)
* Correspondence: weiyan@szu.edu.cn

S1. Image acquisition

Detailed parameters of the image acquired process are as follows:

1. Image of fluorescent beads on the lateral profile: (1) acquisition mode: xyz, (2) scan direction: unidirectional, (3) scan speed: 400Hz, (4) format: 512×512, (5) pixel size (micron): 0.015, (6) frame average: 1, (7) line average: 1, (8) pixel dwell time (microsecond): 1.44.
2. Image of fluorescent beads on 3D: (1) acquisition mode: xzy, (2) scan direction: unidirectional, (3) scan speed: 400Hz, (4) format: 1024×1024, (5) pixel size (micron): 0.018, (6) frame average: 1, (7) line average: 1, (8) pixel dwell time (microsecond): 0.721.
3. Imaging of the lateral nuclear pore complex: (1) acquisition mode: xyz, (2) scan direction: unidirectional, (3) scan speed: 400Hz, (4) format: 1024×1024, (5) pixel size (micron): 0.020, (6) frame average: 1, (7) line average: 1, (8) pixel dwell time (microsecond): 0.721.
4. Image of nuclear pore complexes in 3D: (1) acquisition mode: xzy, (2) scan direction: unidirectional, (3) scan speed: 600Hz, (4) format: 1024×1024, (5) pixel size (micron): 0.021, (6) frame average: 1, (7) line average: 1, (8) pixel dwell time (microsecond): 0.48.

S2. Deconvolution

Deconvolution is performed with the Huygens software (Scientific Volume Imaging). Detailed parameters of the deconvolution process are as follows: (1) background: automatic estimation, (2) estimate mode: lowest, (3) area radius (micron): 0.7, (4) deconvolution algorithm: CMLE, (5) maximum iterations: 10, (6) signal-to-noise ratio: 7, (7) quality threshold: 0.05, (8) iteration mode: optimized, (9) brick layout: auto and (10) PSFs per brick: auto.

S3. Mathematical formula of 3D DE-STED imaging

The PSFs of the donut and the 3D DE-STED images are calculated with the formulas:

$$\mathrm{PSF}_{\mathrm{Donut\ Lateral}} = \mathrm{PSF}_{\mathrm{Confocal\ Lateral}} - \mathrm{PSF}_{\mathrm{STED\ Lateral}} \quad \text{(S1)}$$

$$\mathrm{PSF}_{\mathrm{Donut\ Axial}} = \mathrm{PSF}_{\mathrm{Confocal\ Axial}} - \mathrm{PSF}_{\mathrm{STED\ Axial}} \quad \text{(S2)}$$

where $\mathrm{PSF}_{\mathrm{Confocal\ Lateral}}$, $\mathrm{PSF}_{\mathrm{STED\ Lateral}}$, and $\mathrm{PSF}_{\mathrm{Donut\ Lateral}}$ are the PSFs of confocal, STED, and donut images on the lateral side; $\mathrm{PSF}_{\mathrm{Confocal\ Axial}}$, $\mathrm{PSF}_{\mathrm{STED\ Axial}}$, $\mathrm{PSF}_{\mathrm{Donut\ Axial}}$ are the PSFs of confocal, STED, and donut images on the axial side.

$$\mathrm{PSF}_{\mathrm{DE-STED\ Lateral}} = \mathrm{PSF}_{\mathrm{Confocal\ Lateral}} - \mathrm{K} \times \mathrm{PSF}_{\mathrm{Donut\ Lateral}} \quad \text{(S3)}$$

$$\mathrm{PSF}_{\mathrm{DE-STED\ Axial}} = \mathrm{PSF}_{\mathrm{Confocal\ Axial}} - \mathrm{K} \times \mathrm{PSF}_{\mathrm{Donut\ Axial}} \quad \text{(S4)}$$

where $\mathrm{PSF}_{\mathrm{DE-STED\ Lateral}}$ is the PSF of 3D DE-STED in the lateral direction, $\mathrm{PSF}_{\mathrm{DE-STED\ Axial}}$ is the PSF of 3D DE-STED in the axial direction, and K is the enhancement coefficient.

S4. Calculation of the SNR and PSNR

The SNR and PSNR of the DE-STED images are calculated with the formulas:

$$MSE = \frac{1}{mn}\sum_{i=0}^{m-1}\sum_{j=0}^{n-1}\|f(x,y) - F(x,y)\|^2 \quad (S5)$$

$$SNR = 10 \cdot log_{10}\left[\frac{\sum_{x=1}^{N_x}\sum_{y=1}^{N_y}(f(x,y))^2}{\sum_{x=1}^{N_x}\sum_{y=1}^{N_y}(f(x,y)-F(x,y))^2}\right] \quad (S6)$$

$$PSNR = 10 \cdot log_{10}\left[\frac{\sum_{x=1}^{N_x}\sum_{y=1}^{N_y}(255)^2}{\sum_{x=1}^{N_x}\sum_{y=1}^{N_y}(f(x,y)-F(x,y))^2}\right] = 10 \cdot log_{10}\left[\frac{255^2}{MSE}\right] \quad (S7)$$

where m and n represent the number of pixels in the X-and Y-directions; F(x, y) donates the original confocal image; f(x, y) donates the DE-STED image; and MSE is the mean squared error.

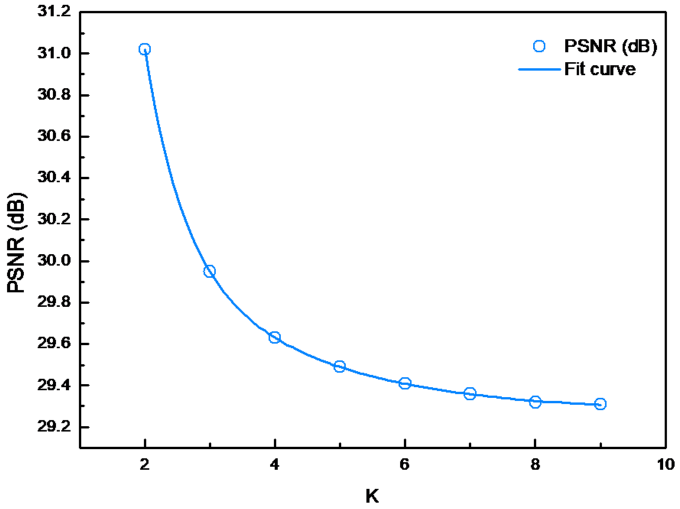

Figure S1. The curves of the relationship between PSNR and the K value of fluorescent beads 3D DE-STED image.
